# A Bibliometric Review of Person-Centered Care Research 2010–2024

**DOI:** 10.3390/healthcare13111267

**Published:** 2025-05-27

**Authors:** Lai-Kun Tong, Hon-Lon Tam, Ai-Mei Mao

**Affiliations:** 1 Research Management and Development Department, Kiang Wu Nursing College of Macau, Macau SAR, China; chingco@kwnc.edu.mo; 2 Nethersole School of Nursing, Faculty of Medicine, The Chinese University of Hong Kong, Hong Kong SAR, China; hltam@cuhk.edu.hk; 3 Education Department, Kiang Wu Nursing College of Macau, Macau SAR, China

**Keywords:** person-centered care, bibliometrics, research trends, thematic evolution

## Abstract

**Background/Objectives**: Person-centered care (PCC) has become a pivotal concept in healthcare. At present, no published studies have assessed the PCC field using bibliometric tools. This study aimed to identify hot spots, trends, and developmental trajectories within the PCC field. **Methods**: Publications related to PCC from 2010 to 2024 were extracted from the Web of Science core collection database and analyzed by the Bibliometrix package from RStudio. **Results**: A total of 5837 studies were analyzed. The analysis revealed steady growth in PCC research, with the United Kingdom, Australia, and the USA leading in publication numbers. Frequent keywords included patient-centered care, PCC, and qualitative research. The thematic shift from patient-centered care to PCC highlights a growing emphasis on individual healthcare needs and values. The evolution of research themes related to PCC has varied across different time periods, with communication, quality improvement, multimorbidity, and chronic disease remaining underdeveloped during 2020–2024, indicating that these themes are key focuses for future research. Emerging keywords over the past five years—value-based healthcare; deep learning; telehealth; and COVID-19—suggest new research directions. **Conclusions**: This study provides a detailed overview of the PCC research landscape, highlighting key areas of focus and identifying potential directions for future research. The findings suggest a dynamic field with a growing emphasis on individualized care and the integration of new methodologies and themes to address current healthcare challenges.

## 1. Introduction

Person-centered care (PCC) is a philosophy and approach to healthcare that emphasizes the customization of healthcare delivery to meet the unique preferences, needs, and values of each patient [1]. It focuses on the whole person, considering physical, emotional, social, and spiritual aspects, to tailor care that aligns with personal goals and values [2]. This approach fosters a partnership between patients and healthcare providers [3], moving away from traditional, provider-centered models that have long dominated the healthcare landscape. PCC has been linked to numerous benefits, including increased patient engagement, empowerment, and advocacy [4,5], as well as improvements in disease management, treatment adherence [6], and organizational efficiency [7]. Moreover, PCC has been shown to reduce patient anxiety [8] and enhance patient satisfaction [9], which are crucial factors in the quality of care. Therefore, numerous healthcare policies and reforms have championed PCC worldwide [10].

Consequently, academic research and scholarly publications in the field of PCC have experienced a notable increase [11]. However, the field continues to exhibit fragmentation, characterized by the diverse range of definitions [12], settings [13,14], approaches, and methodologies [15]. The fragmented nature of the field makes it challenging to identify overarching trends and to synthesize findings across studies. Moreover, the unclear research trajectory of PCC further complicates efforts to advance the field. Understanding the evolution of the field of PCC is essential for guiding future research and policy efforts aimed at enhancing PCC practices. Analyzing the research trajectory helps identify key themes, methods, and the evolving knowledge system within the field. This clarity enables researchers to pinpoint gaps and deficiencies in existing studies, guiding future research directions. Furthermore, it provides policymakers with insights to develop more precise policies that promote the comprehensive implementation and optimization of PCC. Given this context, it is necessary to examine the current state, future trends, and underlying intellectual structure of the field of PCC.

Bibliometric analysis is a systematic research method that uses quantitative analysis of literature to identify patterns, trends, and influences within a specific field [16]. This analytical approach helps identify research gaps and potential research directions and can also help researchers avoid duplication of efforts and ensure efficient use of resources [17]. However, bibliometric analysis on PCC is lacking. In 2007, the World Health Organization introduced a policy framework for PCC [18]. Since 2009, it has emphasized the need to place people at the center of service delivery as part of its core policy directions [19]. In 2010, significant policy initiatives were launched globally to promote PCC. For example, the Chinese Ministry of Health initiated the “Quality Nursing Service Demonstration Project” to enhance clinical nursing services and emphasize patient-centered care. These efforts marked a pivotal moment in the development of PCC, making 2010 a logical starting point for this analysis. In this study, bibliometric analysis was used to analyze literature related to PCC published between 2010 and 2024. This study sought to identify hot spots, trends, and developmental trajectories within the PCC field and point out areas that need further refinement and those that merit further research. It can serve as a foundation for future research, providing researchers with a deeper understanding of PCC and supporting its advancement.

## 2. Materials and Methods

### 2.1. Search Strategy

**Figure 1:**
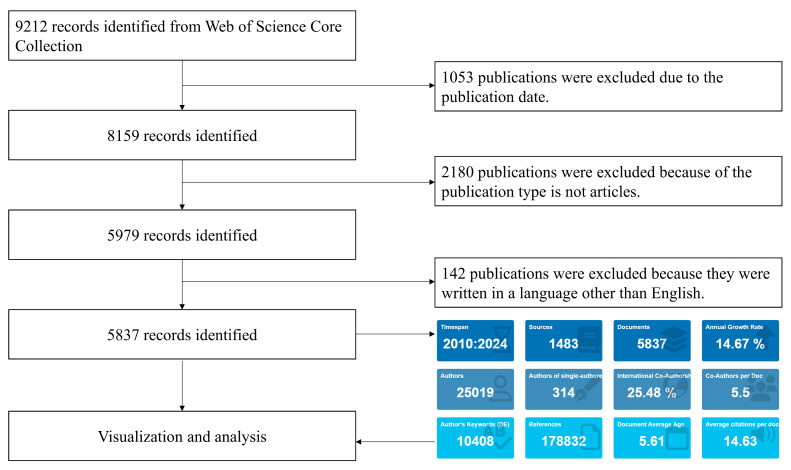
Flowchart for publication screening.

Considering the comparable attributes of bibliometric indicators present in both the Web of Science (WOS) and Scopus databases, the decision was made to utilize WOS due to its more comprehensive citation analysis and broader historical coverage [20], allowing for a more thorough assessment of the documents included [21], with more comprehensive citation analysis and broader historical coverage than Scopus. We used the WOS Core Collection database to collect bibliometric data based on topic-related searches (including title, abstract, and keywords) to ensure optimal positioning of content. The search formula was set to [“client-centered care” (Topic) OR “client-centered practice” (Topic) OR “person-centered care” (Topic) OR “person-centered practice” (Topic) OR “patient-centered care” (Topic) OR “patient-centered practice” (Topic) OR “individualized care” (Topic) OR “people-centered care” (Topic) OR “people-centered practice” (Topic)]. Figure 1 illustrates the process of study screening.

### 2.2. Eligibility Criteria

Literature on PCC was included in this study, ranging from 1 January 2010, to 31 December 2024. Article types included only “articles”, excluding reviews, conference abstracts, letters, expert views, editorial materials, corrections, retractions, and proceedings papers. The publication language was limited to English. Figure 1 shows the process of literature screening.

### 2.3. Data Extraction

The search and data extraction process was conducted by a team of two researchers to ensure reliability and validity. These researchers followed a predefined protocol that included the selection of full records and cited references, ensuring that all relevant bibliographic information was captured. The records were exported and saved as plain text files to maintain data integrity and facilitate subsequent analysis. To minimize bias associated with database updates, the entire extraction was conducted within a single day on 2 January 2025.

### 2.4. Data Analysis

Automated data analysis was performed utilizing the Bibliometrix R-package (version 4.1.4, available at https://www.bibliometrix.org/home/index.php/layout/biblioshiny, accessed on 2 April 2025), which is designed for quantitative research in scientometrics and bibliometrics within the R statistical programming environment [22]. The results were visualized using the Bibliometrix package and Microsoft Excel. Annual scientific production and average citation metrics were obtained to assess the overarching trends in this field. A time trend analysis was conducted using linear regression, with year as the independent variable and the number of annual articles as the dependent variable. This method was selected for its ability to provide a clear and interpretable measure of the overall growth trend in PCC research over the study period. The influential countries, institutions, journals, and authors were primarily evaluated based on their publication output. A chi-square test analyzed the top five countries’ publication counts to determine if there were differences among them. Additionally, established algorithms such as Bradford’s Law, Lotka’s Law, and the H-index were employed to identify core journals and key authors.

The author’s keywords were analyzed by creating a tree diagram of the 50 most common ones and examining the frequency changes of the top 10 over time. A co-occurrence network analysis, designed to uncover keyword relationships, was conducted with settings including automatic layout, association normalization, Walktrap clustering, 50 nodes, removal of isolated nodes, and a minimum of two edges. To further categorize and interpret the keywords, a thematic map was developed. This map divided the keywords into four quadrants based on their centrality (importance within the network) and density (number of connections). The quadrants represent different types of themes: motor themes (high centrality and density), niche themes (low centrality and high density), emerging or declining themes (low centrality and density), and basic themes (high centrality and low density). Additionally, a thematic evolution analysis was conducted to track keyword frequency and co-occurrence over time, providing insights into the evolution of PCC from 2010 to 2024. Thematic mapping and evolution were performed with these settings: 250 words, a minimum cluster frequency of 5, 3 labels, and the Walktrap clustering algorithm.

## 3. Results

### 3.1. Research Trends

**Figure 2:**
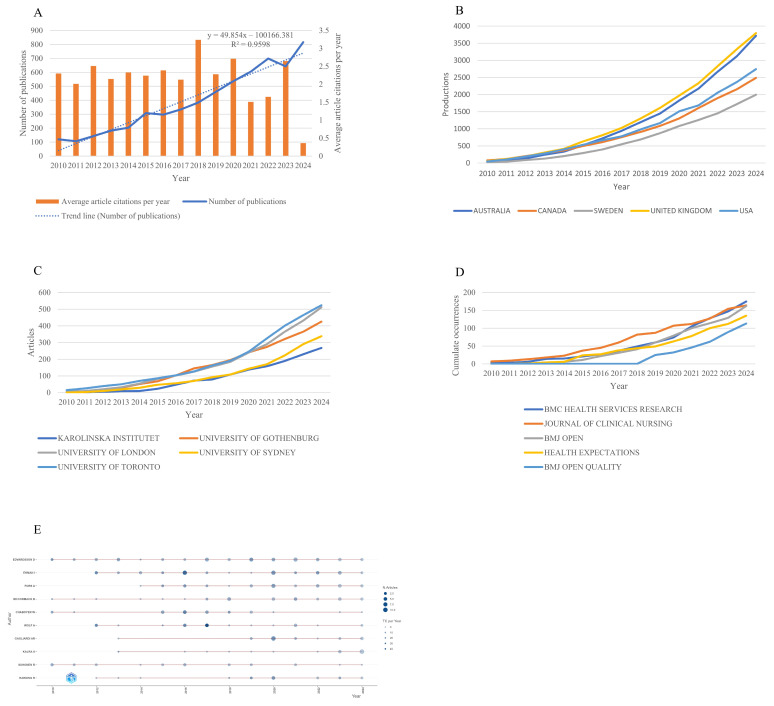
Research trends. (**A**) The annual trends of global publications and citations. (**B**) Top five countries’ production over time. (**C**) Top five affiliations’ production over time. (**D**) Top five sources’ production over time. (**E**) Authors’ production over time. The circle size indicates the number of publications, while the color shade shows the total number of citations.

According to the retrieval process illustrated in Figure 1, a total of 5837 pertinent articles were gathered. The literature concerning PCC exhibited a consistent upward trajectory (y = 49.854x − 100166.381, R^2^ = 0.9598), culminating in a peak of 816 publications in 2024 (Figure 2A). This trend indicates that over the past 15 years, PCC has increasingly garnered the attention of researchers. The average number of citations per paper was 14.63, with the average citations per year reaching a maximum of 3.24 in 2018.

Publications were distributed across 117 countries/regions (Figure S1A), with the top five countries/regions in terms of publication numbers shown in Figure 2B. A chi-square test revealed significant differences among them (χ^2^ = 311, *p* < 0.001). In 2013, the United Kingdom (*n* = 307) surpassed Canada (*n* = 289) in terms of publication output, becoming the country/region with the most publications, a position it has maintained since. In 2016, Australia (*n* = 720) overtook the United States (*n* = 668) to become the second most prolific country/region in terms of publications, a rank it has held ever since, with the United States consistently ranking third thereafter (Figure 2B). Among the countries that collaborated the most on PCC research were the United Kingdom, the United States, and Australia (Figure S1B).

The top 10 institutions in terms of publication output are shown in Figure S1C. Except for the years 2017 and 2019, when the University of Gothenburg ranked first in publication output, it ranked third in all other years, while the University of Toronto consistently ranked first, and the University of London ranked second (Figure 2C). Based on Bradford’s law, the top 10 productive journals were identified as the core journals (Figure S1D). The Journal of Clinical Nursing has been ranked first, with BMC Health Services Research ranking second from 2010 to 2014, but it was surpassed by Health Expectations from 2015 to 2017. In the period from 2019 to 2024, BMJ Open saw a rapid increase in publication output, exceeding Health Expectations.

More than 80% of authors posted only one article in this field by Lotka’s law (Figure S1E). According to the H-index, the top 10 most influential scientists are summarized in Figure S1F. Edvarsson D published annually from 2010 to 2024, being the most prolific author in 2011, 2012, 2013, 2017, 2019, 2021, and 2022 (Figure 2E). Ekman I has been published since 2012, leading in 2012, 2014, 2016, 2020, 2022, and 2023 (Figure 2E). WOLF A published about half the articles of EDVARDSSON D, but WOLF A’s work received more citations. Consequently, WOLF A was the third most influential scientist based on the H-index.

### 3.2. Hotspots Investigation

**Figure 3:**
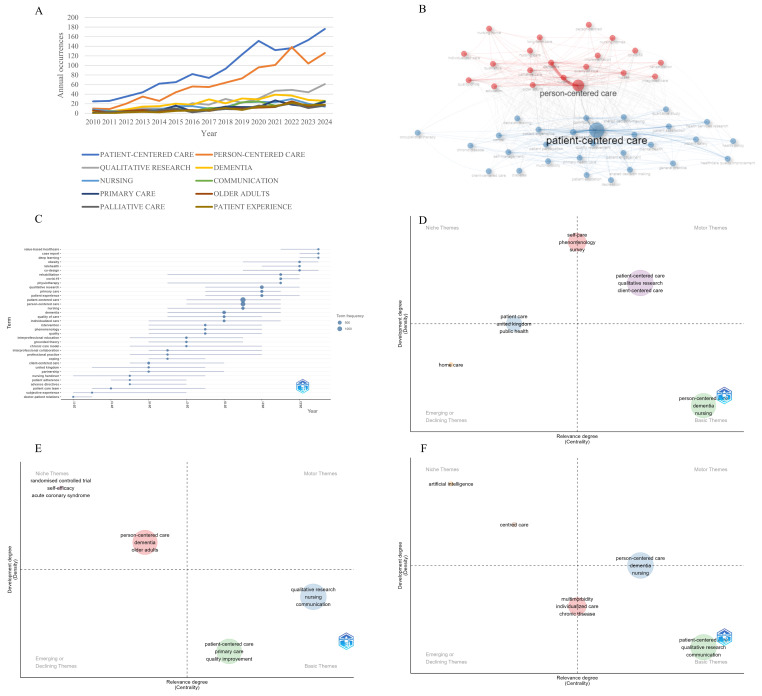
Research hotspots and trends. (**A**) Author’s keywords’ frequency over time. (**B**) Author’s keyword co-occurrence network. The circle size reflects the keyword frequency, while the color indicates the clustering type. Connections show co-occurrence relationships. Nodes of the same color in the same cluster suggest a close relationship. Node size and line width correlate with the strength of co-occurrence. (**C**) Trend topics of author’s keywords. The lines show the period during which each keyword emerged and disappeared, and the dots indicate the peak frequency of each keyword’s mention in publications for each respective year. (**D**) Thematic map: 2010–2014. The thematic map categorizes topics across four quadrants: niche themes are in the upper left, motor themes in the upper right, emerging or declining themes in the lower left, and basic themes in the lower right. (**E**) Thematic map: 2015–2019. The thematic map categorizes topics across four quadrants: niche themes are in the upper left, motor themes in the upper right, emerging or declining themes in the lower left, and basic themes in the lower right. (**F**) Thematic map: 2020–2024. The thematic map categorizes topics across four quadrants: niche themes are in the upper left, motor themes in the upper right, emerging or declining themes in the lower left, and basic themes in the lower right.

Figure S2A illustrates the 50 most frequently occurring author’s keywords within the realm of PCC research. Since 2010, patient-centered care and PCC have persistently occupied the first and second positions, respectively, in terms of frequency among authors’ keywords. The third position has experienced a transition over the years, initially dominated by nursing from 2010 to 2011, followed by dementia from 2012 to 2015, and more recently by qualitative research (Figure 3A).

Our findings revealed a keyword co-occurrence network with two main interconnected clusters (Figure 3B). Cluster 1 (blue) concentrated on patient-centered care, featuring keywords like patient-centered care, communication, and primary care. Cluster 2 (red) focused on PCC, highlighting keywords such as PCC, dementia, palliative care, and quality of care.

The longest-running terms in the author’s keywords were rehabilitation, individualization, chronic care model, professional practice, and nursing handover, which lasted for eight years (Figure 3C). Over the past five years, keywords like value-based healthcare, case report, deep learning, telehealth, and COVID-19 have emerged, indicating a potential new research focus within the field of PCC (Figure 3C). Figure S2B illustrates the thematic evolution from 2010 to 2024, highlighting a shift from patient-centered to PCC, with a focus on holistic individual needs. The diversification of research methods, notably more qualitative studies and randomized controlled trials in more recent years, shows a drive for deeper understanding and robust evidence. Emerging themes like multimorbidity address current healthcare challenges and broaden research areas.

A thematic map of the author’s keywords is divided into four regions based on density and centrality (Figure S2C). The lower right quadrant features themes with high centrality but low density, including patient-centered care, primary care, and quality improvement in cluster 1 and qualitative research, communication, and patient experience in cluster 2. These themes are foundational but underdeveloped. The upper left quadrant includes themes with high density but low centrality, including PCC, dementia, and nursing in cluster 1 and interprofessional education in cluster 2. These themes indicate well-developed but specialized areas. Central topics, including palliative care, qualitative, and quality of care, have intermediate density and centrality, potentially transitioning from foundational to core topics or serving as bridges between different areas.

Between 2010 and 2024, the focus on PCC evolved significantly. From 2010 to 2014, patient-centered care and qualitative research were well-developed, while PCC and dementia were still emerging (Figure 3D). By 2015–2019, these latter themes had matured (Figure 3E), but communication and quality improvement remained underdeveloped, a status that continued into 2020–2024. Additionally, multimorbidity and chronic disease were also underdeveloped themes during 2020–2024 (Figure 3F).

## 4. Discussion

This study aimed to identify the hotspots, trends, and developmental trajectories within the PCC field by analyzing a dataset comprising 5837 publications from 2010 to 2024 using bibliometric methods. Using WOS data, we initially assessed publication and citation trends over the years, as well as the contributions of various countries, institutions, journals, and authors. Additionally, bibliometric tools were employed to identify authors’ keywords, thereby elucidating research hotspots and trends.

This study’s results highlight a steady 15-year increase in PCC literature, underscoring its growing significance in healthcare, as also noted in earlier studies [23,24]. This trend is supported by evidence that PCC enhances healthcare quality and benefits both patients and providers [25]. Consequently, there is a rising interest in PCC from researchers, policymakers, and politicians, reflected in its adoption across diverse healthcare settings [10,26,27]. However, this study shows that PCC research is heavily concentrated in high-income economies, indicating extensive study and implementation in these regions. These affluent nations, particularly in North America and Europe, have the resources to support personalized care models and have led in adopting PCC practices, focusing on patient satisfaction and outcomes [28]. The disparity in research between high-income and lower-income countries calls for a more equitable global health research approach. This would make the benefits of PCC available to all populations, regardless of economic status [29].

This study indicates that since 2010, both patient-centered care and PCC have been prominent keywords, with a notable thematic shift towards PCC from 2010 to 2024. This shift reflects a growing emphasis on individual needs and values in healthcare. From 2010 to 2014, patient-centered care received considerable focus, highlighting the importance of patient involvement in medical decisions to enhance satisfaction and care quality [5,30,31]. However, this model was criticized for its clinical focus and failure to address comprehensive individual needs [32]. Since 2015, PCC has risen in prominence, offering a more holistic approach that considers an individual’s environment, social relations, emotions, and cultural background [33]. This model has been particularly significant in psychiatry, where it has replaced the traditional disease-centered model by respecting patient autonomy and preferences, leading to more comprehensive health support [34,35]. PCC has also been applied to long-term and home care, improving chronic disease management and quality of life, and to multimorbidity, enhancing health outcomes through coordinated care [35,36]. The transition from patient-centered care to PCC between 2010 and 2024 marks a shift towards recognizing the holistic needs of individuals. While patient-centered care focuses on functional life, PCC focuses on a meaningful life for individuals [2]. This evolution underscores the healthcare system’s growing priority on humanistic care and personalized services, moving towards a more integrated and humane healthcare environment.

In the past five years, emerging keywords like value-based healthcare, deep learning, telehealth, and COVID-19 have indicated new research directions in PCC. As global healthcare demand rises due to an aging population and increasing chronic diseases, along with escalating costs, value-based healthcare has become crucial [37,38]. This approach focuses on enhancing medical service quality and efficiency to control costs and improve patient outcomes, aligning with PCC goals [39,40,41]. It integrates healthcare services, improves payment models, and advances information technology to boost medical care quality [42,43]. Modern information technology supports value-based healthcare implementation. The COVID-19 pandemic has spurred telehealth and digital health tool use, changing healthcare delivery and promoting research [44,45]. These technologies offer new methods for PCC [46], helping providers meet personalized patient needs and increase engagement and satisfaction [47,48,49]. However, challenges exist [50], such as data privacy [51], digital divides [52], and maintaining care quality [53]. To harness these technologies’ potential in PCC, service models must be continuously optimized. This includes robust data security frameworks, equitable access to digital tools, and training for healthcare providers. Consequently, integrating telehealth and digital health into PCC is now a key research area, potentially reshaping future healthcare delivery.

This bibliometric analysis from 2010 to 2024 reveals significant shifts in PCC research focus. From 2010 to 2014, well-developed themes like patient-centered care and qualitative research formed a solid base for understanding patient experiences and qualitative aspects of care. Concurrently, PCC and dementia were emerging interests, although not fully integrated into research. The application of PCC in dementia care was limited by a lack of unified definitions and standards [54], leading to early adoption in only a few regions, such as the United Kingdom [55]. Broader acceptance began post-2014 [56]. Therefore, between 2015 and 2019, there was a notable maturation of the themes of PCC and dementia, reflecting a deeper integration of these concepts into healthcare practices and research agendas [57,58]. However, communication and quality improvement remained underdeveloped, indicating a gap in research that could be critical for the effective implementation of PCC. This underdevelopment persisted into the period from 2020 to 2024, suggesting a continued need for focused research to address these areas.

Additionally, the themes of multimorbidity and chronic disease were also identified as underdeveloped during 2020–2024. Despite the WHOs endorsement of PCC, several obstacles hinder its study among multimorbid patients: (1) A lack of specialized tools for multimorbid patients [59], (2) Challenges in implementing PCC, including interdisciplinary collaboration and complexity [60], and (3) A gap between the theoretical and practical aspects of PCC among healthcare professionals [61]. These issues are particularly pertinent as the prevalence of multimorbidity and chronic disease rises in aging populations [62,63] and the potential of PCC to improve outcomes in such contexts [13]. The underdevelopment of these themes highlights challenges in integrating care models for patients with multiple chronic conditions. The evolving research focus on PCC over time underscores the dynamic nature of healthcare research and the need for adaptation to new challenges. This emphasizes the need for targeted research in communication, quality improvement, multimorbidity, and chronic disease management within PCC. The bibliometric analysis from 2010 to 2024 provides an overview of PCC research, showing progress and areas needing attention. Future research should address these gaps to enhance the effectiveness of PCC, improving patient outcomes and healthcare efficiency.

## 5. Practical Implications

The bibliometric analysis of PCC research from 2010 to 2024 offers valuable insights for healthcare professionals, educators, and policymakers. The persistent focus on rehabilitation, individualization, chronic care models, professional practice, and nursing handover underscores their significance in PCC and the necessity for ongoing enhancement and integration in clinical practice. The recent emergence of keywords such as value-based healthcare, deep learning, and telehealth reflects a move toward more innovative, technology-driven PCC approaches. Healthcare professionals are encouraged to adopt telehealth and digital health tools to boost patient engagement and optimize care delivery. Educators should incorporate these emerging themes into training programs to equip future healthcare professionals with the skills needed for effective PCC implementation. The ongoing underdevelopment of themes like multimorbidity and chronic disease highlights the need for targeted research and policy initiatives to address these critical gaps.

## 6. Limitation

While this study provides valuable insights into the landscape of PCC through a bibliometric analysis, several limitations should be acknowledged. Firstly, the study relied solely on the WOS Core Collection database for data collection. While WOS is a widely used database, it may not encompass all pertinent literature, potentially resulting in an incomplete portrayal of the PCC research landscape. Furthermore, relying only on database indexing without manually searching article references could have omitted studies from non-indexed or gray literature, leading to significant studies being excluded. Secondly, the study period from 2010 to 2024 was chosen to capture recent trends and developments in PCC research. However, this timeframe may exclude earlier foundational work that has significantly contributed to the field. Thirdly, there are inevitably some relevant articles that were overlooked since the search terms were limited. Future research should consider broader search terms and strategies to ensure a more comprehensive capture of the literature in this evolving field. Fourthly, our analysis is based solely on English-language publications, which may introduce language bias. This limitation could potentially exclude valuable contributions from non-English-speaking regions, thereby narrowing the scope of our findings. Future studies may benefit from including a broader range of languages to capture a more comprehensive view of the global research landscape in PCC. Fifthly, the interpretation of causality from bibliometric data presents inherent challenges. Bibliometric analysis provides insights into research trends, publication patterns, and knowledge structures, but it does not directly establish causal relationships. The observed correlations and trends should be interpreted with caution, as they may be influenced by various factors, including changes in research funding, policy priorities, and technological advancements. Future research should consider additional qualitative and quantitative methods to explore the underlying causes and implications of the identified trends. Finally, the interpretation of bibliometric indicators, such as citation counts and H-index, should be approached with caution. These metrics can be influenced by various factors, including publication practices, citation behaviors, and the visibility of journals. Therefore, while bibliometric indicators provide useful insights, they should not be the sole basis for evaluating the impact or quality of research.

## 7. Conclusions

This study analyzed PCC research published from 2010 to 2024, summarizing key aspects including regional and institutional distribution, collaboration networks, journal and author analysis, citation metrics, keyword clustering, thematic mapping, and trend topics. Despite a growing focus and predicted steady growth in PCC research over the past 15 years, output remains largely concentrated in high-income economies. The thematic shift from 2010 to 2024 from patient-centered care to PCC highlights a growing emphasis on individual healthcare needs and values. The evolution of research themes related to PCC has varied across different time periods, with communication, quality improvement, multimorbidity, and chronic disease remaining underdeveloped during 2020–2024, indicating that these themes are key focuses for future research. Emerging keywords over the past five years—value-based healthcare, deep learning, telehealth, and COVID-19—suggest new research directions. This study offers valuable insights into current PCC research, aiding researchers in identifying emerging trends and hotspots.

## Data Availability

The original contributions presented in this study are included in the article/Supplementary Materials. Further inquiries can be directed to the corresponding author.

## Supplementary Materials

The following supporting information can be downloaded at: https://www.mdpi.com/article/10.3390/healthcare13111267/s1, Figure S1: Quantitative analysis of publication; Figure S2: Hotspots investigation of publication.

## Abbreviations

The following abbreviations are used in this manuscript:

PCC	Person-centered care
WOS	Web of Science
WHO	World Health Organization
