# Supplementary material for: A Bibliometric Review of Person-Centered Care Research 2010–2024
Source: Healthcare (Basel). 2025 May 27;13(11):1267. doi: 10.3390/healthcare13111267 (PMC12154729; doi:10.3390/healthcare13111267)
Supplement: Supplementary file 1 [file healthcare-13-01267-s001.zip › healthcare-3600240-supplementary.pdf]

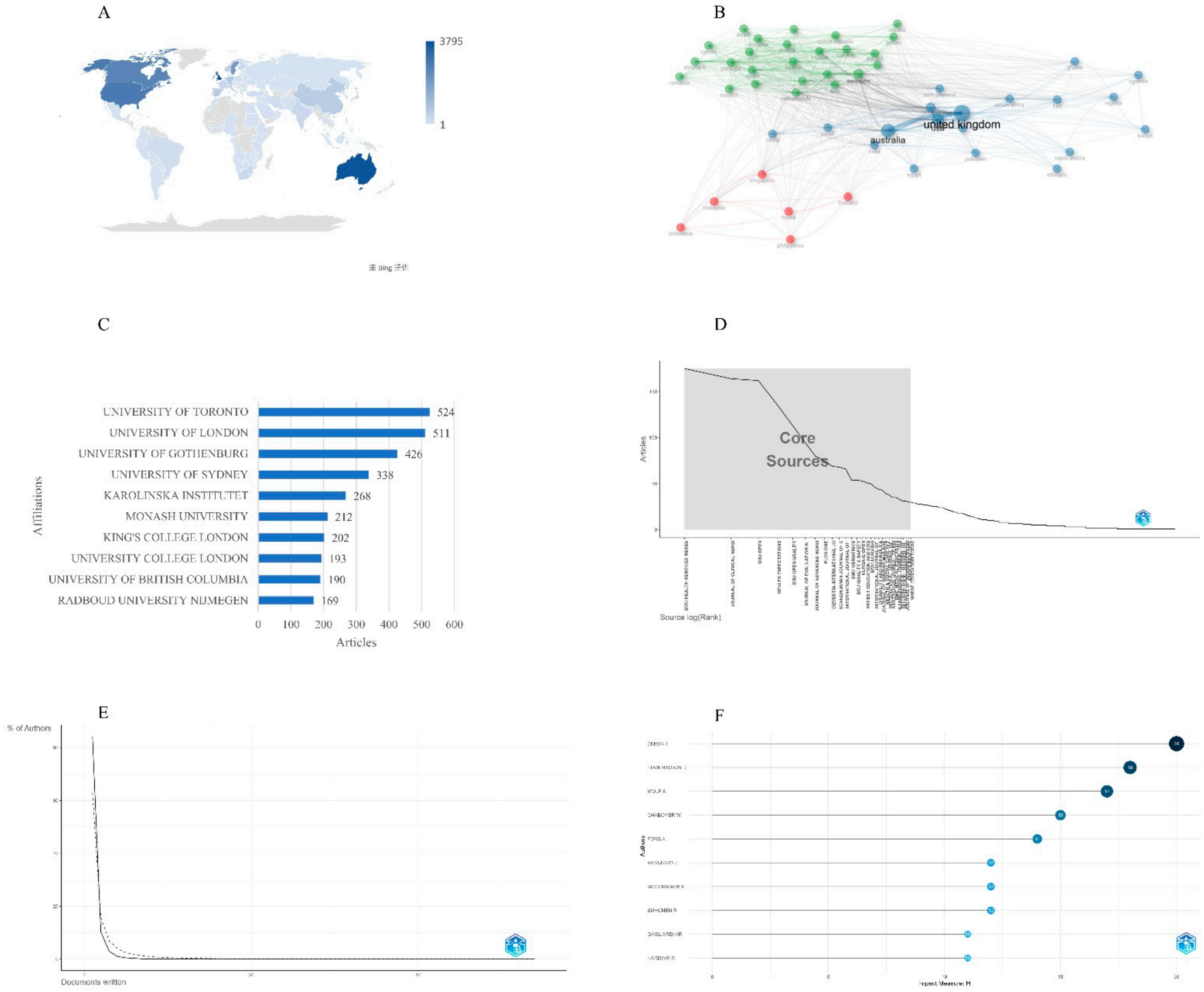

Figure S1 Quantitative analysis of publication. (A) Countries' scientific production. (B) Collaboration among countries/regions. (C) Most relevant affiliations. (D) Core sources by Bradford's Law. (E) Author productivity through Lotka's Law. (F) Authors' local impact.

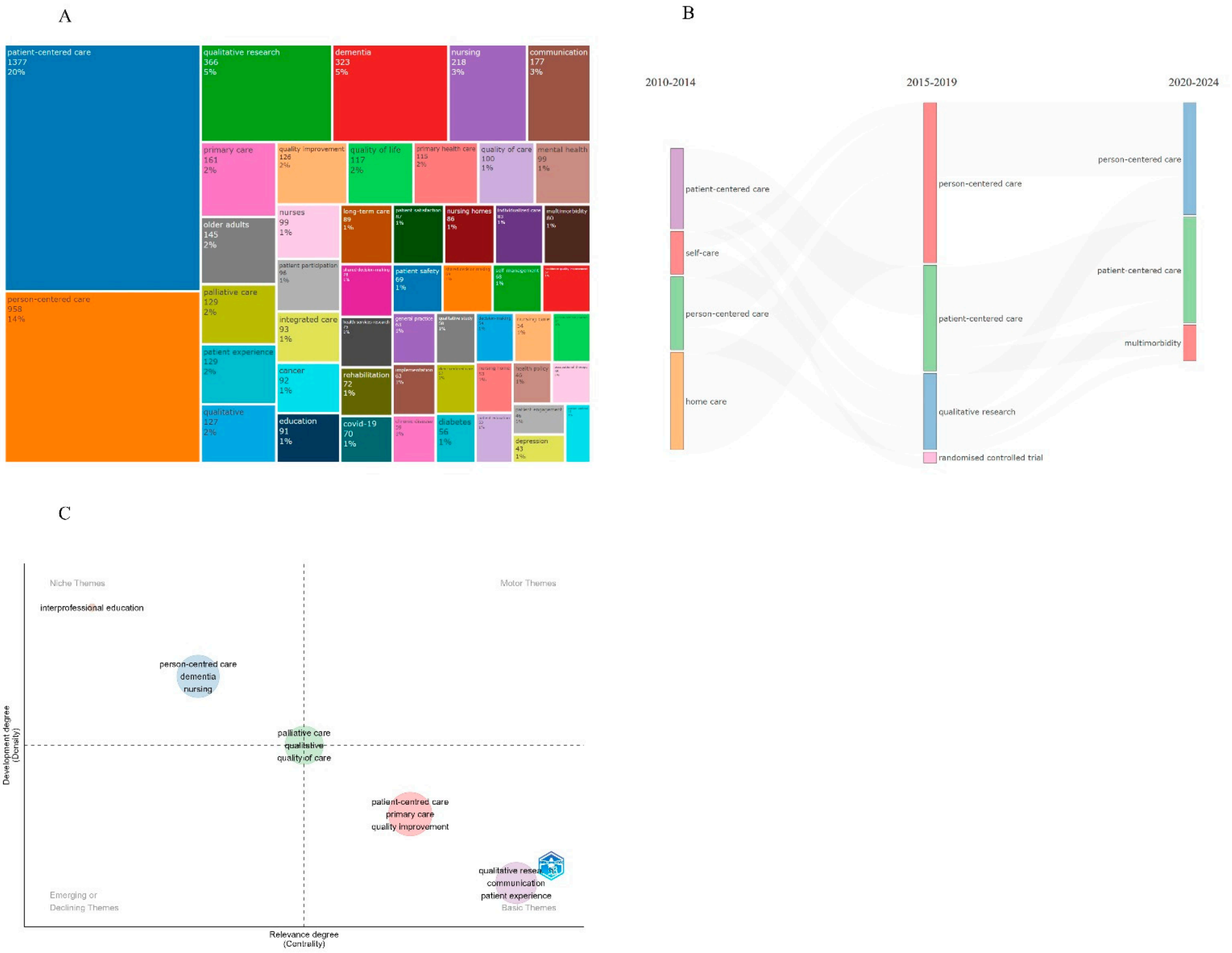

Figure S2 Hotspots investigation of publication. (A) Keywords TreeMap. (B) Thematic evolution. (C) Thematic map.
